# The Development and Use of Reporter Influenza B Viruses

**DOI:** 10.3390/v11080736

**Published:** 2019-08-09

**Authors:** Rebekah E. Dumm, Nicholas S. Heaton

**Affiliations:** 1 Department of Molecular Genetics and Microbiology, Duke University School of Medicine, Durham, NC 27710, USA; 2 Department of Molecular Genetics and Microbiology (MGM), Duke University Medical Center, 213 Research Drive, 426 CARL Building, Box 3054, Durham, NC 27710, USA

**Keywords:** Influenza B virus, reverse genetics, reporter virus, molecular virology, viral genetic engineering

## Abstract

Influenza B viruses (IBVs) are major contributors to total human influenza disease, responsible for ~1/3 of all infections. These viruses, however, are relatively less studied than the related influenza A viruses (IAVs). While it has historically been assumed that the viral biology and mechanisms of pathogenesis for all influenza viruses were highly similar, studies have shown that IBVs possess unique characteristics. Relative to IAV, IBV encodes distinct viral proteins, displays a different mutational rate, has unique patterns of tropism, and elicits different immune responses. More work is therefore required to define the mechanisms of IBV pathogenesis. One valuable approach to characterize mechanisms of microbial disease is the use of genetically modified pathogens that harbor exogenous reporter genes. Over the last few years, IBV reporter viruses have been developed and used to provide new insights into the host response to infection, viral spread, and the testing of antiviral therapeutics. In this review, we will highlight the history and study of IBVs with particular emphasis on the use of genetically modified viruses and discuss some remaining gaps in knowledge that can be addressed using reporter expressing IBVs.

## 1. Introduction to Influenza B Virus and Associated Disease

Influenza B viruses (IBVs) are negative-sense, segmented RNA viruses of the Orthomyxoviridae family [1]. IBV was first discovered in 1940 and described as “a new type of virus from epidemic influenza” [2]. At that time, it is thought that only IBV strains from the “Yamagata” lineage were circulating. In 1983 however, Rota et al. described the emergence of a new lineage of IBV, which was named the “Victoria” lineage [3]. Since at least 1983, both the Yamagata and Victoria lineages have circulated in the human population, with varying prominence in different influenza seasons [4].

IBV infection causes acute respiratory disease including the induction of the innate immune response which is associated with fever, body aches, and fatigue; a set of symptoms collectively termed “influenza-like illness” [5,6]. Primary viral infection causes significant damage to the pulmonary epithelial tissue, inducing vulnerability to disease complications [7,8,9,10,11]. Secondary bacterial infections [7] or, especially in cases of preexisting lung disease, life-threatening acute respiratory distress syndrome [11], are examples of complications that account for significant IBV induced morbidity and mortality [12,13,14]. Although IBVs substantially contribute to the total burden of influenza disease [15,16], IBVs have received disproportionately less investigation and research funding than the related influenza A viruses (IAVs). Therefore, we currently have an incomplete understanding of IBV pathogenic mechanisms and potential approaches to mitigate disease.

The historical lack of focus on IBVs is likely due to an incomplete understanding of the prevalence and disease severity of IBV. Until recently, influenza subtypes isolated from human clinical infections were differentiated on the basis of hemagglutination inhibition testing, a process that lacks sensitivity [17]. It wasn’t until 2010 that PCR sequencing technology was available to quickly determine the relative amounts of IBV strains [18]. Implementing this technology has increased our understanding of its prevalence, and it is now thought that IBV is responsible for 15–30% of total influenza disease and 11–27% of hospitalizations (as measured by the CDC influenza surveillance, seasons 2010–2018, avg 23.4% [19]). Our under-appreciation of IBV-induced disease has also been influenced by out-of-date publications which report the disease as mild [20,21]. Recent studies quantifying the severity of IBV infections have reported that when factors such as hospitalization rates, lengths of stay, and economic impact are examined, there were no significant differences between IBV- and IAV-induced disease [15,22,23,24,25]. Influenza C and D viruses, while distinct from IAVs and IBVs, also cause acute respiratory disease. Influenza C virus induces mild disease in humans [26,27,28] and influenza D virus predominantly circulates in swine, cattle, and sheep [29,30]. These viruses are not monitored in routine clinical or epidemiological studies due to the low incidence of clinically-relevant human infections [16,19].

**Figure 1:**
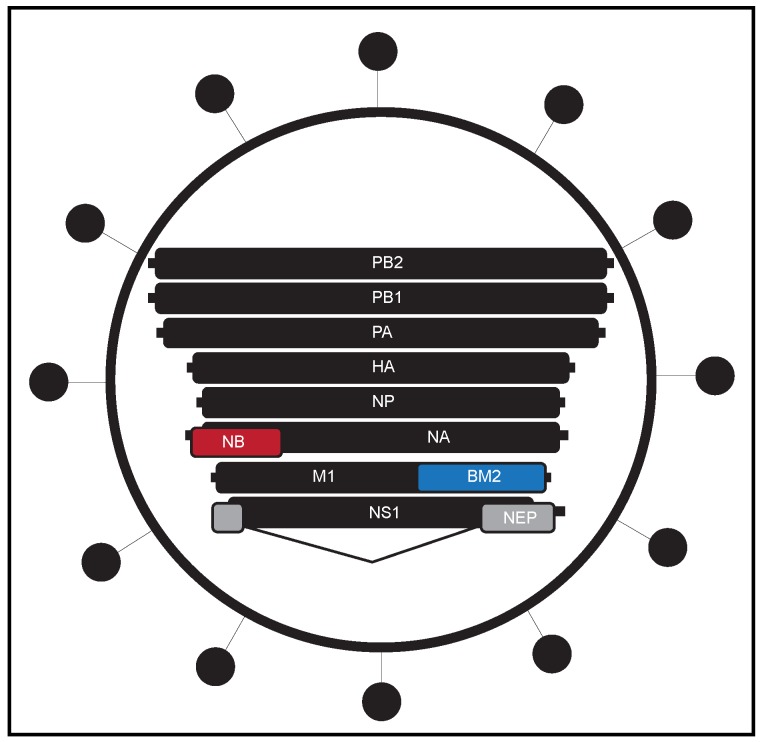
Schematic illustrating unique influenza B virus proteins. The RNA genome of the segmented influenza B virus (not to scale) with the unique gene products (relative to other influenza viruses) shown in color. Abbreviations: *PB2*—polymerase basic protein 2, *PB1*—polymerase basic protein 1, *PA*—polymerase acidic protein, *HA*—hemagglutinin, *NP*—nucleoprotein, *NB*—glycoprotein NB, *NA*—neuraminidase, *M1*—matrix protein, *BM2*—BM2 protein, *NS1*—non-structural protein 1, *NEP*—nuclear export protein.

While IAV and IBV both induced significant disease burden in humans, there are fundamental differences between IAV and IBV, some of which have been described [31,32,33,34,35,36], and others which remain unknown [37]. One of the most significant distinctions is that IBV has not been shown to have an established animal reservoir [32], with only rare reports reporting human strains of IBV found in seal and porcine species [38,39,40]. Additionally, IBV has not been reported to undergo reassortment (which in IAVs can lead to pandemic outbreaks), but instead relies on antigenic drift to drive viral evolution [41] more similar to seasonal IAV strains [42]. IBV also encodes unique genes not expressed by IAV, such as *NB* and *BM2* (Figure 1), and even among shared proteins, there is relatively little sequence identity [31,32].

Genetic differences between the influenza viruses have the potential to drive a range of genera specific characteristics. For example, IBVs have been shown to interact differently with the human host compared to IAV. Specifically, viral tropism in the respiratory tract for IBVs can be distinct from IAVs, influenced by strain-specific hemagglutinin (HA) structures and differential affinities for host glycans [33,34]. Differences have also been reported with respect to the kinetics of innate immune activation and interferon signaling that occur after IAV vs. IBV infection [35,36]; the potential impact of these differences, however, remains unclear. Genera specific differences can also affect vaccine production; IBVs (in contrast to IAVs) have traditionally not grown well in embryonated chicken eggs, leading to poor vaccine yield and emergence of mutations that can affect antigenicity [43,44].

Our current understanding of IBV biology and mechanisms of pathogenesis, though incomplete, are derived from a number of different scientific approaches. Previous studies have used “mini-genome” replicase assays to study aspects of the viral life cycle related to replication [45,46]. The use of tagged proteins and functional mutants have contributed to the study of protein–protein interactions [47,48]. The use of semi-infectious viruses has allowed the study of viral RNA packaging signals [49,50]. Finally, high throughput sequencing and novel modeling techniques for studying viral phylogeny have been used to better understand both viral drift and evolutionary dynamics [51,52] as well as improving surveillance and informing vaccine decisions [52,53]. In this review, we will focus on another important experimental approach, the use of genetically modified IBVs. We will briefly outline the history of genetically modified IBVs with particular emphasis on reporter expressing strains, describe recent insights derived from their use, and finally discuss how their future use will continue to facilitate the study of IBV biology.

## 2. Development of an Influenza B Virus Reverse Genetics System

Currently, we can make use of viral reverse genetic approaches to generate viral tools that allow us to define how influenza viruses interact with their host and cause disease. Multiple fundamental discoveries, however, were crucial in enabling the development and use of these systems [32,54]. Due to the segmented nature of the influenza virus genome and the lack of mapping of the complete viral genome, viruses generated solely from cloned DNA remained elusive until the late 1990s [55,56]. Throughout the 1980s and 1990s, there were a series of discoveries made regarding the structure of the IBV genome that helped build the foundation for future genetic tools. In 1978, Skehel and Hay discovered that the terminal portions of each segment, the untranslated regions (UTRs), are conserved [57]. Not only are these regions conserved among IBVs, but the UTRs of IBVs are unique and much longer on the 5′ end than those of IAVs [56]. Also, these non-coding terminal sequences were shown to be essential for viral replication [55], and complete mapping of these terminal sequences was necessary to allow genetic manipulation of IBVs.

**Figure 2:**
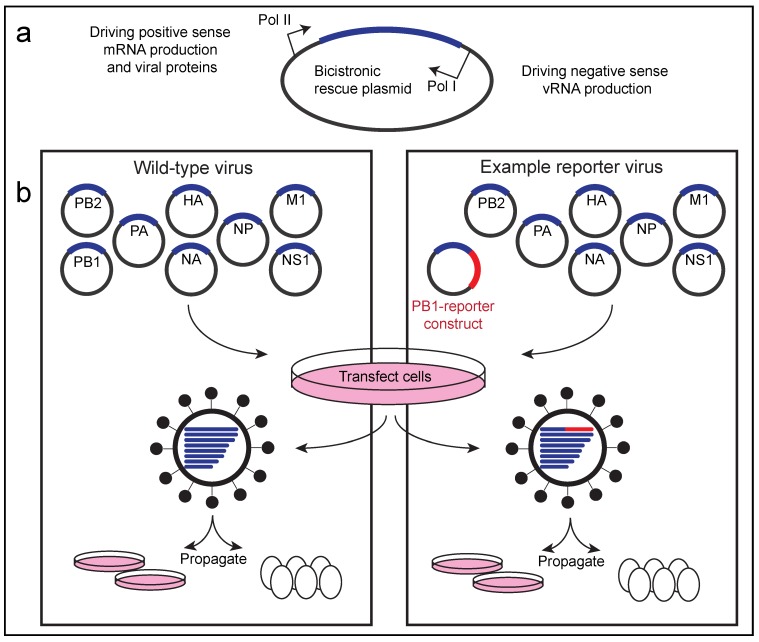
Schematic outlining influenza virus reverse genetic systems. (**a**) Bi-cistronic plasmid that drives expression of positive-sense viral mRNA for gene transcription and negative-sense viral RNA for genomic replication. (**b**) Transfection of an eight-plasmid system (one plasmid per viral genomic segment) allows production of recombinant wild-type virus. Alternatively, genomic segment plasmids containing reporter constructs can be introduced in place of wild-type (WT) segments to generate viruses harboring reporter genes.

In 1999, the first reports of reverse genetics systems for IAVs were published [58,59]. Using similar approaches, two groups published the first IBV recombinant genetic systems three years later [60,61]. Generally, these recombinant systems include at least eight separate plasmids (one for each segment of the influenza genome) which utilize RNA polymerase I (Pol I) promotors to drive transcription of negative-sense viral RNA. To produce the viral replicase proteins (consisting of NP, PB1, PB2, and PA) needed to replicate and transcribe the vRNA, Pol II promoters in the opposite orientation of the Pol I promoters, are also required (Figure 2). The first rescue of IBV from cDNA was in 2002, when Hoffmann et al. reported the rescue of the strain B/Yamanashi/166/98 [60]. Not only did they rescue the recombinant virus, but they also substituted the influenza glycoproteins, HA and NA, for the glycoproteins of B/Victoria/504/200, B/Hawaii 10/2001, and B/Hong Kong/330/2001, demonstrating that the glycoproteins have some level of interchangeability, which is especially relevant for vaccine manufacturing [60]. Shortly following this report, Jackson et al. described a virus that was rescued in the background of B/Beijing/1/87 [61].

Since the development of the IBV reverse genetic system, several groups have used genetically modified viruses to study IBV biology, highlighting the unique features such as the *NB, BM2* genes expressed by IBV. Starting in 2003, Hatta et al. reported that the influenza NB protein, an accessory protein encoded on segment 6 that is not conserved in IAVs, could be knocked out and therefore shown to be unnecessary for B/Lee/40 viral replication in vitro [62]. Further studies have since demonstrated that this protein does not affect replication or transmission in in vivo models [63].

It was also during the years following the introduction of reverse genetics for IBV that the function for the IBV BM2 protein, which is encoded on segment 7, was discovered. In 2004, several groups used reverse genetic systems to demonstrate that BM2 was necessary for incorporation of viral ribonucleoprotein complexes into virions and replication [64,65]. Additional studies were done to elucidate the translational mechanisms of BM2 [66,67], ultimately revealing the use of a termination/re-initiation strategy [68].

Mutant viruses have also been used to probe the mechanisms of innate immune evasion of IBVs. In the B/Lee/40 viral background, it was discovered that the nonstructural protein (NS1) was essential for viral growth. These studies demonstrated that, despite the lack of protein homology between IAV NS1 and IBV NS1, the interferon antagonizing functions are conserved [69,70]. Later work described the immune interaction of IBV NS1 with the host effectors (protein kinase R, phosphatidylinositol 3-kinase) which are crucial effectors in the interferon-response pathway that are differentially activated during IAV infection [71,72,73]. Finally, several studies have described the nuances of host immune interactions, demonstrating that IBV NS1 inhibition of interferon-stimulated genes is species-specific [74,75]. These studies all linked viral genes or portions of genes to features of viral infection and pathogenesis, experiments made possible by the development of the reverse genetic system for IBVs.

## 3. Development of Reporter Influenza B Viruses

Although the IBV genome could be genetically modified as early as the late 1990s, there was a lack of viral strains that harbored reporter proteins for rapid and sensitive detection of viral infection. Hatta et al. were the first group to insert reporter genes into the IBV genome, albeit at the expense of the virus losing full replication potential. In this work, the authors found that the IBV neuraminidase (NA) open-reading frame (ORF) could be replaced by green fluorescent protein (GFP) and viral spread could occur if the requirement for neuraminidase enzymatic activity was satisfied via addition of exogenous sialidases [65].

**Figure 3:**
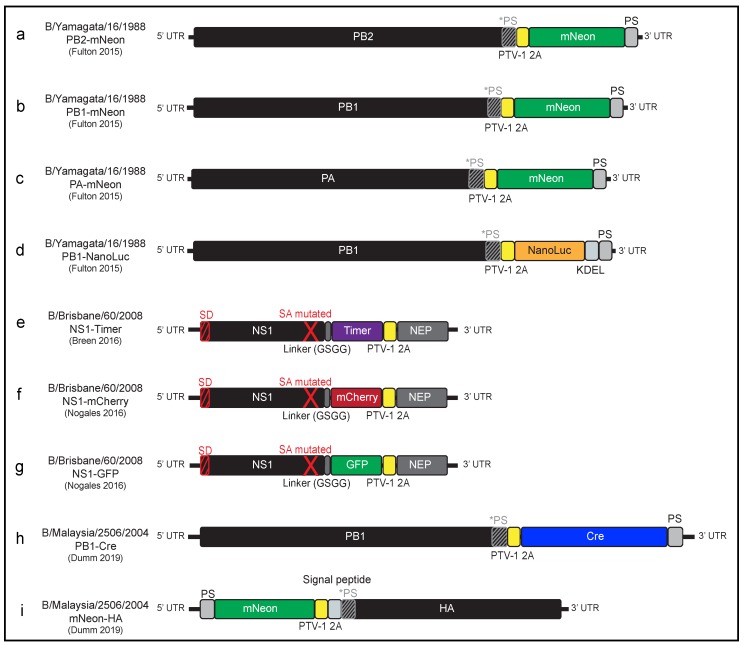
Designs of published influenza B virus genomic segments harboring reporter genes. (**a**–**c**) mNeon expressed in each of the polymerase subunit segments (**d**) NanoLuc expressed in the PB1 segment (**e**–**g**) Fluorescence proteins expressed in the NS1/NEP segment (**h**) Cre recombinase expressed in the PB1 segment (**i**) mNeon expressed in the HA segment. Abbreviations: PB2—polymerase basic protein 2, PB1—polymerase basic protein 1, PA—polymerase acidic protein, HA—hemagglutinin, NS1—non-structural protein 1, NEP—nuclear export protein. GFP—green fluorescent protein, UTR—untranslated region, *PS—silently mutated packaging signal, PS—duplicated packaging signal, PTV-1 2A—porcine teschovirus 2A sequence for co-translational separation, KDEL—endoplasmic reticulum retention sequence to prevent secretion, SD—Splice donor site, SA mutated—mutated Splice acceptor site.

In order to develop fully infectious reporter IBV strains, knowledge from the development of reporter IAVs [76,77,78,79,80,81] was applied to IBVs. The first report of a replication-competent IBV demonstrated the insertion of an exogenous protein in the B/Yamagata/16/1988 background in 2015 [82]. In this paper, Fulton et al. showed that the insertion of an mNeon reporter protein was tolerated in each of the polymerase segments of the genome. These mNeon gene insertions were accomplished by encoding the reporter gene after a 2A protease motif [83,84] following the viral protein ORFs, and adding a segment specific packaging signal after the reporter coding region (Figure 3a–c). These viruses allowed fluorescent quantification of multicycle growth in vitro, and as a proof of principle for screening drug compounds, the authors measured the inhibitor effect of the drug Zanamizir, which inhibits the activity of viral neuraminidase. Similarly, Fulton et al. inserted NanoLuc, a bioluminescent protein, into the PB1 segment and were able to monitor growth via luciferase activity (Figure 3d). Finally, the authors utilized this NanoLuc-expressing virus to test neutralizing antibodies and found improvement over traditional methods of quantification [82].

Shortly after this report, Breen et al., designed and rescued a replication-competent reporter IBV which contained a Timer fluorescent reporter which undergoes a spectral shift over the course of time [85]. In the NS1 segment of B/Brisbane/60/2008, they disrupted the splicing donor/acceptor sites and duplicated the NEP gene to allow expression of both proteins (NS1 and NEP) in addition to the Timer reporter protein (Figure 3e). They used this tool to measure the rate and spread of viral infection in vitro [85]. Shortly after the NS1-Timer strategy was published, this same group published a second study describing a similar design [86]. In this report, Nogales et al. inserted mCherry and GFP into the NS1 segment of B/Brisbane/60/2008 (Figure 3f–g) and demonstrated that these viruses could be used for high-throughput screening approaches for antivirals (Ribavirin and Amantadine) and polyclonal antibodies [86].

Most recently, Dumm et al. published a paper using the B/Malaysia/2506/2004 background to express Cre recombinase in the PB1 segment of the genome (Figure 3h). The authors were then able to infect cells containing a lox-stop-lox-reporter cassette both in vitro and in vivo to permanently label infected cells and measure the survival of cells after direct viral infection [87]. Using this tool, Dumm et al. showed that ciliated cells in the upper respiratory tract survive direct infection with IBV. In addition to surviving infection, the cells became both transcriptionally and phenotypically distinct from uninfected ciliated cells. As part of this study, a reporter virus expressing mNeon in the HA segment of the genome (Figure 3i), was also generated and found to accurately report on viral infection both in vitro and in vivo [87].

The viruses described in these reports all have advantages and limitations based on their specific design and the reporter protein expressed (described in Table 1). For example, viruses expressing fluorescent proteins allow real-time detection of viral replication without additional fixing and staining steps. Similarly, luciferase-expressing viruses allow for detection of viral infection with excellent sensitivity. However, the viral genomic modifications that allow the development of these reporter viruses can affect viral biology, and care must be taken to ensure that experimental interpretations from experiments with reporter viruses also extend to the unmodified parental strains. For example, the fusion of reporter proteins or exogenous peptides (i.e., the remaining 21 amino acids of the 2A protease motif) to viral proteins frequently the virus and may disrupt protein–protein interactions. Additionally, without extensive stability testing, multicycle growth in eggs or in vivo experiments can result in loss of the reporter gene and misinterpretation of phenotypes.

## 4. Gaps in Influenza B Virus Knowledge That Can Be Addressed Using Reporter Viruses

The recombinant viral tools generated in the last 20 years have facilitated many important discoveries, however important scientific questions remain. Some of the most pressing concerns in the influenza field are the design of effective vaccines to prevent disease and the discovery of additional antiviral therapeutics. While modification of the virus itself to function as a better vaccine has been reviewed elsewhere [32,54,61], reporter viruses represent promising tools for rapid, sensitive readouts of vaccine efficacy. As demonstrated by some of the reports above [45,65,82,85,86,87], reporter viruses expressing fluorescent or bioluminescent proteins can facilitate the measurement of neutralizing antibody efficacy and the effects of antiviral compounds. In order to best facilitate these studies, the future development reporter strains in contemporary, clinically-relevant circulating strains of IBV are of high importance.

There are also basic science questions which remain to be answered. IBV generally presents as an infection of the upper respiratory tract. However, a feature which results in severe influenza disease is lower respiratory tract involvement leading to acute respiratory distress syndrome [11]. Much of the research of this complication has been in the context of IAV disease [11,88], however reports of IBV instigating this more severe disease are not uncommon [13,14,15,22,89]. New reporter IBVs could help to elucidate the mechanisms by which viral disease progresses into this life-threatening condition. Specifically, the use of viruses encoding reporters that emit in the infrared spectrum, as has been utilized in other experimental systems [90,91,92], would allow non-invasive and longitudinal measurements of viral dissemination across the respiratory tract. These viruses would be relevant for a variety of pathogenesis model systems and could greatly inform our understanding of how to approach cases of severe disease or even mild cases with high-risk of progressing to poor outcomes.

Finally, there are fundamental aspects of viral biology, ranging from the functions of viral proteins to the mechanisms controlling viral assembly and reassortment that remain incompletely characterized for influenza viruses in general [93]. Research to address some of these questions has begun using advanced microscopy techniques and deep sequencing [94,95,96,97]. Replication-competent reporter viruses however, are also valuable tools to ask these types of questions. For example, genetically modified viruses adding tags or modifications to the viral genomic segments or protein, can allow precise and rapid experimental readouts during reassortment studies [98,99,100]. Additionally, while a variety of reporter viruses have been made for IAV [101], there are several proteins of IBV (NB, BM2) [6] that are not produced by IAV and therefore will require specific tools to better understand their functions. By generating tagged or reporter protein fused versions of these proteins in a fully infectious viral background, one can monitor protein subcellular localizations and trafficking in real time during infection.

In conclusion, genetically modified and reporter viruses add to the molecular virologists’ toolbox and have already been used to answer important questions about IBV biology. More work is necessary, however, to expand the available virus repertoire and engineer novel viruses to increase our growing understanding of IBV and facilitate the development of next generation vaccines and antiviral therapeutics.

## Figures and Tables

**Table 1:** Overview of published replication-competent reporter influenza B viruses and their strengths, limitations, and corresponding references.

Reporter Virus	Strengths	Limitations	Author, Year, Ref
**B/Yamagata/16/1988 PB1-mNeon**	Bright reporter expression (4 log_10_ signal increase vs background) Fluorescent signal detectable by 12 hpi Endpoint titer same as parental virus Reporter gene stable after 4 passages	Growth kinetics delayed vs parental virus In vivo characterization not reported 2A site leaves 21 aa on C-term of PB1 (GSGATNFSLLKQAGDVEENPG)	Fulton, 2015, [82]
**B/Yamagata/16/1988 PB2-mNeon**	Bright reporter expression (4 log_10_ signal increase vs background)	Endpoint viral titer not reported Growth kinetics not reported Reporter stability not reported In vivo characterization not reported 2A site leaves 21 aa on C-term of PB2	Fulton, 2015, [82]
**B/Yamagata/16/1988 PA-mNeon**	Detectable reporter expression (3 log_10_ signal increase vs background)	Endpoint viral titer not reported Growth kinetics not reported Reporter stability not reported In vivo characterization not reported 2A site leaves 21 aa on C-term of PA	Fulton, 2015, [82]
**B/Yamagata/16/1988 PB1-NanoLuc**	High levels of reporter expression (6 log signal increase vs background) Luciferase signal correlates with infection over 5 log_10_ viral titration	Endpoint titer lower and growth kinetics delayed vs parental virus Reporter stability not reported In vivo characterization not reported 2A site leaves 21 aa on C-term of PB1	Fulton, 2015, [82]
**B/Brisbane/60/2008 NS1-Timer**	Fluorescent spectral shift of Timer protein tracks dynamics of IBV infection Fluorescent signal detectable by 8 hpi No decrease in NP expression relative to parental virus	Endpoint titer lower and growth kinetics delayed vs parental virus Reporter gene 61.2% retained after 5 passages In vivo characterization not reported 2A site leaves 1 aa on N-term of NEP Timer fused to C-term of NS1 Splicing of NS1-NEP disrupted	Breen, 2016, [85]
**B/Brisbane/60/2008 NS1-mCherry**	NS1-mCherry fusion protein allows visualization of NS1 localization Fluorescent signal detectable by 18 hpi Endpoint titer and growth kinetics same as parental virus NP expression same as parental virus	Reporter stability not reported In vivo characterization not reported 2A site leaves 1 aa on N-term of NEP Splicing of NS1-NEP disrupted	Nogales, 2016, [86]
**B/Brisbane/60/2008 NS1-GFP**	NS1-GFP fusion protein allows visualization of NS1 localization Fluorescent signal detectable by 18 hpi	Endpoint titer lower and growth kinetics delayed vs parental virus Reporter stability not reported In vivo characterization not reported 2A site leaves 1 aa on N-term of NEP Splicing of NS1-NEP disrupted	Nogales, 2016, [86]
**B/Malaysia/2506/2004 PB1-Cre**	Endpoint titer same as parental virus Reporter gene stable after 4 serial passages	Growth kinetics delayed vs parental Virus attenuated in mouse model vs parental virus 2A site leaves 21 aa on C-term of PB1	Dumm, 2019, [87]
**B/Malaysia/2506/2004 mNeon-HA**	Detectable reporter expression (3 log_10_ signal increase vs background) after in vivo infection	Endpoint viral titer not reported Growth kinetics not reported Reporter stability not reported Virus attenuated in mouse models 2A site leaves 1 aa on HA signal peptide	Dumm, 2019, [87]
